# Calibration and Validation of Machine Learning Models for Physical Behavior Characterization: Protocol and Methods for the Free-Living Physical Activity in Youth (FLPAY) Study

**DOI:** 10.2196/65968

**Published:** 2025-04-16

**Authors:** Samuel Robert LaMunion, Paul Robert Hibbing, Scott Edward Crouter

**Affiliations:** 1 Diabetes, Endocrinology, and Obesity Branch - Energy Metabolism Section National Institute of Diabetes, Digestive, and Kidney Diseases National Institutes of Health Bethesda, MD United States; 2 Department of Kinesiology, Recreation, and Sports Studies The University of Tennessee Knoxville Knoxville, TN United States; 3 Department of Kinesiology and Nutrition The University of Illinois Chicago Chicago, IL United States

**Keywords:** physical behavior assessment, youth activity, wearable devices, activity monitoring, digital health, physical behavior characterization, criterion dataset

## Abstract

**Background:**

Wearable activity monitors are increasingly used to characterize physical behavior. The development and validation of these characterization methods require criterion-labeled data typically collected in a laboratory or simulated free-living environment, which does not generally translate well to free-living due to limited behavior engagement in development that is not representative of free living.

**Objective:**

The Free-Living Physical Activity in Youth (FLPAY) study was designed in 2 parts to establish a criterion dataset for novel method development for identifying periods of transition between activities in youth.

**Methods:**

The FLPAY study used criterion measures of behavior (direct observation) and energy expenditure (indirect calorimetry) to label data from research-grade accelerometer-based devices for the purpose of developing and cross-validating models to identify transitions, classify activity type, and estimate energy expenditure in youth aged 6-18 years. The first part of this study was a simulated free-living protocol in the laboratory, comprising short (roughly 60-90 s) and long (roughly 4-5 min) bouts of 16 activities that were completed in various orders over the span of 2 visits. The second part of this study involved an independent sample of participants who agreed to be measured twice (2 hours each time) in free-living environments such as the home and community.

**Results:**

The FLPAY study was funded from 2016 to 2020. A no-cost extension was granted for 2021. A few secondary outcomes have been published, but extensive analysis of primary data is ongoing.

**Conclusions:**

The 2-part design of the FLPAY study emphasized the collection of naturalistic behaviors and periods of transition between activities in both structured and unstructured environments. This filled an important gap, considering the traditional focus on scripted activity routines in structured laboratory environments. This protocol paper details the FLPAY procedures and participants, along with details about criterion datasets, which will be useful in future studies analyzing the wealth of device-based data in diverse ways.

**International Registered Report Identifier (IRRID):**

RR1-10.2196/65968

## Introduction

Device-based assessment of physical behavior has become common in health research [1-6]. Despite significant advancements in this field, a major challenge remains: the translation and generalization of device outputs, which are crucial for accurate interpretation and use [1,7]. This challenge arises partly from the methodological approaches used, as well as from the way data are collected to calibrate and validate these methods. Traditional static regression models for physical behavior assessment, such as those used in the 2003-2006 National Health and Nutrition Examination Survey [8], rely on population-, attachment site-, and device-specific cut points or regression equations to estimate energy expenditure (EE) and physical activity intensity [9]. However, these models tend to perform poorly when applied to the dynamic and complex nature of real-world behavior, as well as when tested on out-of-sample datasets [10,11]. For example, legacy models such as the Freedson equation for children [12] have been shown to underestimate moderate-to-vigorous physical activity by up to 51% during free-living conditions [11], highlighting a key limitation in the field. This discrepancy arises because these models were developed using structured laboratory data, which fail to capture the sporadic and irregular movement patterns typical of free-living environments. Furthermore, traditional methods often segment accelerometer data into fixed time intervals (eg, 1-min epochs), assuming that each data point is independent and represents a continuous bout of activity—an assumption that is rarely valid in real-world settings [13-15].

Advancements in sensor technology, including the use of wrist-worn accelerometer-based devices and the integration of additional sensor types (ie, gyroscopes and magnetometers), have improved the potential ability to capture a broader range of movement behaviors with different characteristics, such as the discrimination of sedentary behavior [16-18]. These innovations offer promising solutions for improving physical behavior assessment, particularly in youth populations. However, challenges remain in using raw data from these devices to accurately predict EE and categorize activity types [1,19,20]. The use of machine learning algorithms has been an interesting development in this space, showing potential for improving activity classification and EE estimation [21-24]. However, as with traditional methods, these machine learning algorithms have been primarily trained on structured laboratory activities, leading to poor performance when applied in free-living conditions and to new datasets [21,25]. The key issue lies in the segmentation of activity bouts and the identification of transitions between activities, which are much more subtle and irregular in free-living environments compared to the abrupt transitions typical of laboratory-based protocols [26-28].

The Free-Living Physical Activity in Youth (FLPAY) study was an effort to address these gaps by collecting transition-rich data in free-living conditions for calibration and validation of device-based models of physical behavior. The purpose of this paper is to describe the methods and data associated with the FLPAY study, thereby encouraging procedural replication in future studies and providing a comprehensive background for future analyses of data from this study. By addressing the complexities of free-living data, particularly the segmentation of activity bouts and transitions, this study aims to improve the accuracy, precision, and applicability of device-based physical behavior assessments and contribute to the development of more effective methods for characterizing physical behavior in youth.

## Methods

### Study Design

The FLPAY study was a 2-part investigation to develop and validate sensor-based methods for detecting transitions between activities as a precursor to predicting EE. The first part of this study took place in a simulated free-living laboratory environment conducive to method development (calibration), while the second took place in unstructured free-living environments conducive to method validation. Both studies were for youth participants aged 6-18 years who were recruited through word of mouth and flyers. The overall study design was conceived to address common limitations of prior method development studies, which have focused on the collection of steady-state laboratory data and structured ambulatory activities, rather than allowing naturalistic transitions and data collection in free-living.

Below, we present protocol and data summaries for each component of this present study in separate subsections. Portions of the information have been reported previously [26,29-31]. This present study was not a clinical trial. Due to the overlap in the methods for each part of this study (eg, similar equipment), the following sections are focused primarily on unique elements of each part, while recurring elements are presented in Multimedia Appendix 1.

### Ethical Considerations

Both parts of this study were approved by the University of Tennessee Knoxville Institutional Review Board (UTK IRB-15-02487-FB). For all participants, written informed consent was obtained from a parent. Participants also provided written informed assent. Both parts of this study involved video recording on an opt-in basis, and thus, there were separate consent and assent signatures for general enrollment and video recording. However, for both parts of this study, the consent and assent documents did not include language for public data sharing, and thus, only deidentified data will become available from the FLPAY study. For screening, parents filled in a health history questionnaire, with participants being excluded if they had medical conditions or musculoskeletal injuries that prevented engagement in physical activities. Additional exclusion criteria included conditions or medications affecting metabolism. Participants were compensated for their participation in this study. Details on compensation are included within the subsections for each study component.

### Part 1: Laboratory-Based Component

#### Participants—Laboratory-Based Component

A total of 100 participants were recruited. Approximately even distributions were recruited across sex and age groups (6-9, 10-12, 13-15, and 16-18 years). Participant characteristics are shown in Table 1.

**Table 1:** Physical characteristics of participants from the laboratory study.

			Full sample (N=100)		Male (n=48)		Female (n=52)
**Age (years)**							
	Mean (SD)	12.1 (3.53)		12.3 (3.32)		11.9 (3.74)	
	Median (IQR)	12.1 (9.3-15.2)		12.4 (9.9-15)		11.7 (9-15.2)	
**Age group (years), n (%)**							
	6-9	33 (33)		13 (27.1)		20 (38.5)	
	10-12	25 (25)		15 (31.3)		10 (19.2)	
	13-15	23 (23)		12 (25)		11 (21.2)	
	16-18	19 (19)		8 (16.7)		11 (21.2)	
**Height (cm)**							
	Mean (SD)	150 (19.2)		152 (20.5)		148 (17.9)	
	Median (IQR)	151 (134-166)		156 (140-167)		150 (133-162)	
**Sitting height (cm)**							
	Mean (SD)	74.9 (9.5)		75.4 (9.7)		74.3 (9.5)	
	Median (IQR)	74.5 (66.5-83.1)		74.5 (68.6-84)		74.4 (65.9-82.6)	
**Leg length (cm)**							
	Mean (SD)	75 (11.4)		77 (11.8)		73.2 (10.8)	
	Median (IQR)	76 (66-82.8)		77.5 (69.6-85.8)		74.3 (63.8-80)	
**Weight (kg)**							
	Mean (SD)	44.7 (18.7)		46.5 (20.3)		43 (17.2)	
	Median (IQR)	40.5 (29.6-55.7)		41 (34.1-56.3)		38.3 (29.3-55.7)	
**Fat mass (kg)**							
	Mean (SD)	10.4 (7.5)		9.2 (6.8)		11.4 (7.9)	
	Median (IQR)	7.5 (5.7-12.1)		7 (5.6-10)		9.5 (6.5-13.7)	
**Body fat (%)**							
	Mean (SD)	21.9 (6.4)		18.7 (4.8)		24.9 (6.3)	
	Median (IQR)	20.8 (17.5-24.5)		17.5 (15.6-19.7)		23.7 (21.1-27.5)	
**Fat-free mass (kg)**							
	Mean (SD)	34.3 (12.9)		37.2 (14.6)		31.6 (10.6)	
	Median (IQR)	32.7 (23.3-42.8)		33.8 (27.3-48.1)		30.3 (22-41.6)	
**BMI (kg/m^2^)**							
	Mean (SD)	19 (4.4)		19.1 (4.4)		19 (4.5)	
	Median (IQR)	18 (16.2-20.5)		18 (16.3-19.9)		17.9 (16.2-20.6)	
**BMI percentile (%)**							
	Mean (SD)	51.6 (27.4)		50.9 (26.8)		52.3 (28.2)	
	Median (IQR)	49.4 (33.6-73.3)		48.8 (34.2-72.2)		49.6 (33.6-74.6)	
**BMI classification**							
	Underweight	5 (5)		2 (4.2)		3 (5.8)	
	Healthy weight	80 (80)		40 (83.3)		40 (76.9)	
	Overweight	9 (9)		2 (4.2)		7 (13.5)	
	Obese	3 (3)		3 (6.3)		0 (0)	
	Severe obese	3 (3)		1 (2.1)		2 (3.8)	
**Race or ethnicity**							
	Hispanic White	4 (4)		1 (2.1)		3 (5.8)	
	Non-Hispanic African American	9 (9)		3 (6.3)		6 (11.5)	
	Non-Hispanic White	87 (87)		44 (91.7)		43 (82.7)	
**Handedness**							
	Right	94 (94)		45 (93.8)		49 (94.2)	
	Left	6 (6)		3 (6.3)		3 (5.8)	

#### Laboratory-Based Component Procedures

All research visits were conducted in the Applied Physiology Laboratory and the surrounding area at the University of Tennessee Knoxville. The protocol entailed 2 visits on separate days, each lasting approximately 2-2.5 hours. The main feature of the protocol was the completion of 16 activities (Table S3 in Multimedia Appendix 1) that were divided across the 2 visits. A key goal of this study’s design was to simulate realistic transitions between activities. Therefore, each activity was performed twice, with 1 instance lasting approximately 60-90 s and the other lasting approximately 4-5 min. Within those ranges, participants self-selected when they transitioned to the next activity unless guidance was needed from the research team (eg, if the upper limit of 90 s or 5 min was reached or if the walking circuit reached a specific stopping point to facilitate speed calculations). They also self-selected the order of the 8 activities they were prescribed each day, except that the same activity could not be performed twice in a row, and the activities were grouped by location (ie, in an upstairs laboratory or a separate recreation area) to minimize total transition time. The order of short and long bouts for each activity was randomized.

Throughout each research visit, participants wore a portable indirect calorimeter and a variety of activity monitors. Participant behavior and posture were coded by direct observation, using a focal sampling approach that allowed continuous logging of behaviors, transitions, and posture. Video recording was also performed for most participants. The video recordings served as a backup in case the live coded observation records needed to be reviewed and revised for any reason (eg, adjusting timestamps if an error was noted during the live coding process).

#### Laboratory-Based Component Detailed Timeline

For the first visit, participants were asked to refrain from eating or drinking (except water) for 2 hours before the visit and to wear lightweight athletic clothing and closed-toed shoes. After completing the enrollment and consent processes, they removed their shoes and socks and had anthropometric measurements taken. Standing and sitting height (cm) were measured using a wall-mounted stadiometer. Body mass (kg) and body fat (%) were measured using a Tanita BC-418 bioelectrical impedance analyzer (Tanita Co. of America, Inc).

Following the anthropometric measurements, participants were fitted with this study’s equipment and completed a 30-minute assessment of resting metabolic rate (RMR). The remainder of the visit involved completing the 8 activities assigned for that day. Participants received a US $30 gift card at the end of the visit.

For the second visit, participants were given the same previsit instructions. Upon returning to the laboratory, they were fitted with the same study equipment as visit 1 before performing the remaining 8 activities, such that all participants completed the full set of 16 activities under normal circumstances (ie, assuming there is no withdrawal, refusal, or space unavailability due to scheduling). Participants received a US $45 gift card at the end of the visit.

#### Laboratory-Based Component Equipment

All participants wore 5 ActiGraph GT9X devices (GT9X; ActiGraph LLC), which were the primary activity monitors of interest for this study. One was placed on the right hip, 1 on each wrist, and 1 on each ankle. The hip-worn device was worn on an elastic belt and positioned over the right iliac crest along the anterior axillary line and secured using a manufacturer-specific belt clip. The wrist-worn devices were worn in manufacturer-specific watch bands that the devices clipped into. The watch bands were positioned proximal to the styloid processes with the devices oriented where the LCD could be read naturally by the participant, as a standard watch face would be. The ankle-worn devices were clipped into manufacturer-specific clips worn on slip-through Velcro bands. The clips were positioned so that the devices were on the lateral aspect of the ankle proximal to the lateral malleolus. Additional detail is given in Section S1.1 in Multimedia Appendix 1.

Secondary activity monitors, both research- and consumer-grade, were also worn in various locations on the body as detailed in Sections S1.2 and S1.3 in Multimedia Appendix 1.

#### Criterion Measures

Participants were observed using the Noldus Observer XT and Pocket Observer software platforms (Noldus Information Technology), which served as a criterion measure of behavior and posture. Participants also wore a Cosmed K*4b^2^* (COSMED s.r.l.), which was the criterion measure of EE. General details of these tools can be found in Sections S1.4 and S1.5 in Multimedia Appendix 1. Specific details applicable to part 1 of this study are discussed below.

The Observer XT desktop platform (version 12.5) was used to create a 2-class focal sampling coding scheme, which was then exported for use in the Noldus Pocket Observer application (version 3.2). The latter application was run on Samsung Galaxy Tab 4 tablets (Samsung) and used for real-time coding of participant behavior and posture. Several activity monitoring studies have taken a similar approach to direct observation using Noldus products [22,28,32-34].

The coding scheme included a 19-item list of activity behaviors (ie, for the 16 activities listed in Table S3 [Multimedia Appendix 1], plus labels for the start time of the observation period, transitions between activities, and unknown activities) and a 6-item list of postures (lying, reclining, sitting, standing, stepping, or unknown). The label for unknown activities was used whenever an unlisted activity occurred, such as a water break or bathroom break, and was accompanied with a posture label of unknown.

Whenever a new activity was initiated, users tapped the corresponding label in Pocket Observer, which issued a timestamp for the new activity and then prompted the user to indicate the participant’s posture. This created a continuous and timestamped record of activities and postures. After finishing the protocol, live-coded observations were imported back into the Noldus Observer XT software program for conversion to Excel format. The resulting files were then processed in R (R Foundation).

Section S1.5 in Multimedia Appendix 1 describes the procedures that were used when syncing data from the various sources.

#### Processing of Criterion Data for Laboratory-Based Component

While the activity monitor data were collected for flexible use (eg, to be processed and tested in different ways across future studies), the criterion data from direct observation and indirect calorimetry were designed for consistent usage across future studies. Therefore, we describe the processing and aggregation of criterion data in the sections that follow, while monitor data processing is left for future studies to describe according to the unique needs of the study.

#### Direct Observation Data

Each participant was observed by a single trained observer in real time during each study visit. The observation files were inspected for comments and anomalies that indicated errors (eg, the user entered a comment indicating a button was pressed at the wrong time or that the activity duration was outside the expected range). Corrections were made by a senior reviewer cross-referencing the video recordings and adjusting labels or timing accordingly. For participants without video recordings, observations were used as they were created from the live coded direct observation (n=3). Each row of the observation files indicated a start time and duration for the corresponding activity and posture, and by definition, there were no gaps between activities. Thus, every second of the data collection period could be mapped to a specific activity and posture label that was active at that time, allowing a second-by-second record to be constructed from the information in the observation files.

#### Portable Indirect Calorimeter Data

EE was calculated based on oxygen consumption (VO_2_) and RMR, both measured by the K4b^2^. The values were expressed in youth metabolic equivalents (MET_y_), where




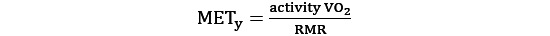




As described in the Sections S2.1 to S2.3 in Multimedia Appendix 1, calculations for RMR and continuous VO_2_ were the same for parts 1 and 2 of the FLPAY study. Below, we provide additional detail about calculating steady state EE as this was unique to part 1 of this study.

Steady state periods were defined for the longer activity bouts. All bouts longer than 3 minutes, 40 seconds were included in the analysis. The steady state period was defined as the last 60 seconds of each activity, after discarding the final 10 seconds. For each of these periods, the corresponding K4b^2^ data were extracted in breath-by-breath format. An average VO_2_ was then calculated using manufacturer-specified procedures (Section S2.1 in Multimedia Appendix 1). This value was divided by RMR to obtain MET_y_ for the steady state period. Posture was determined by cross-referencing the direct observation data from the steady state period.

Activity intensity was derived from EE (MET_y_) and posture, using the following accepted definitions [35]: for sedentary behavior, ≤1.50 MET_y_ and seated, lying, or reclining posture; for light physical activity, 1.51-2.99 MET_y_ regardless of posture (or ≤1.50 MET_y_ if posture was upright); and for moderate-to-vigorous physical activity, ≥3.00 MET_y_ regardless of posture. For each activity and age group (ie, those aged ≤12 versus >12 years), data were cleaned by removing values <0.2 MET_y_, or >2 SD above or below the mean.

### Part 2: Unstructured Free-Living Study

#### Unstructured Free-Living Study Participants

A total of 84 participants were recruited to this study, of whom one was excluded (final N=83). Participant characteristics are shown in Table 2.

**Table 2:** Physical characteristics of participants from the free-living study.

		Full sample (N=83)	Male (n=39)	Female (n=44)
**Age (years)**				
	Mean (SD)	11.2 (3.4)	11.1 (3)	11.3 (3.7)
	Median (IQR)	10.6 (8.5-13)	10.5 (8.9-12.7)	11.2 (8.4-13.6)
**Age group (year), n (%)**				
	6-9	35 (42.2)	17 (43.6)	18 (40.9)
	10-12	26 (31.3)	13 (33.3)	13 (29.5)
	13-15	14 (16.9)	6 (15.4)	8 (18.2)
	16-18	8 (9.6)	3 (7.7)	5 (11.4)
**Height (cm)**				
	Mean (SD)	146 (19.4)	145 (16.1)	147 (22.2)
	Median (IQR)	144 (132-162)	143 (135-159)	145 (130-164)
**Sitting height (cm)**				
	Mean (SD)	73.5 (9.2)	73.3 (8.4)	73.7 (10)
	Median (IQR)	73 (67-80)	73 (67-79.5)	73 (66-80.3)
**Leg length (cm)**				
	Mean (SD)	72.5 (10.9)	72 (8.3)	72.9 (12.8)
	Median (IQR)	71 (66-81)	70.5 (67.3-78.9)	71.8 (63.4-84.9)
**Weight (kg)**				
	Mean (SD)	42 (18.6)	39.9 (13.9)	43.9 (22)
	Median (IQR)	36.1 (28.4-53.9)	36.1 (30.7-50)	37.6 (26.5-55.2)
**Fat mass (kg)**				
	Mean (SD)	8.9 (7.8)	9.7 (6.6)	8.2 (8.8)
	Median (IQR)	6.2 (3.9-11.8)	8.6 (4.3-13.4)	4.8 (3.7-7.5)
**Body fat (%)**				
	Mean (SD)	19.3 (9.3)	21.9 (9.1)	17.1 (9)
	Median (IQR)	17.9 (13.6-24)	22.2 (15.4-27.1)	15.3 (12.6-18.3)
**Fat-free mass (kg)**				
	Mean (SD)	33.1 (13.5)	30.2 (8.2)	35.7 (16.5)
	Median (IQR)	30.2 (23.3-39.7)	27.6 (23.7-37.5)	31.7 (21.9-42.8)
**BMI (kg/m^2^)**				
	Mean (SD)	18.7 (4.3)	18.3 (3.5)	19.1 (4.9)
	Median (IQR)	17.7 (15.8-19.7)	17.8 (15.9-19.6)	17.6 (15.7-20)
**BMI percentile (%)**				
	Mean (SD)	53.3 (26.2)	51.5 (24)	55 (28.2)
	Median (IQR)	52.8 (33.4-72.4)	47.6 (33.7-72.3)	54.5 (33.9-72.5)
**BMI classification, n (%)**				
	Underweight	3 (3.6)	1 (2.6)	2 (4.5)
	Healthy weight	67 (80.7)	34 (87.2)	33 (75)
	Overweight	6 (7.2)	2 (5.1)	4 (9.1)
	Obese	4 (4.8)	1 (2.6)	3 (6.8)
	Severe obese	3 (3.6)	1 (2.6)	2 (4.5)
**Race or ethnicity, n (%)**				
	Hispanic Hawaiian or Pacific Islander	1 (1.2)	0 (0)	1 (2.3)
	Hispanic White	2 (2.4)	2 (5.1)	0 (0)
	Latino White	1 (1.2)	0 (0)	1 (2.3)
	Non-Hispanic African American	2 (2.4)	0 (0)	2 (4.5)
	Non-Hispanic Asian	6 (7.2)	1 (2.6)	5 (11.4)
	Non-Hispanic Native American or Alaskan	1 (1.2)	0 (0)	1 (2.3)
	Non-Hispanic White	70 (84.3)	36 (92.3)	34 (77.3)
**Handedness, n (%)**				
	Left	3 (3.6)	0 (0)	3 (6.8)
	Right	80 (96.4)	39 (100)	41 (93.2)

#### Unstructured Free-Living Study Procedures

The overall goal of this part of this study was to collect free-living data with diverse activities and transitions between them. Therefore, research visits were conducted in varied settings, such as in the community, participants’ homes, local parks, and approved facilities of community partners. Data were collected across 2 separate days, with the goal to have one in the participant’s home and the other somewhere else.

The first study visit was generally conducted in the participant’s home environment and began with the informed consent and assent process and participant screening. Once enrolled, participants had anthropometric measurements taken, including standing and sitting height (cm) using a portable stadiometer and body mass (kg) and body fat percentage measured using a Tanita BF-350 bioelectrical impedance analyzer. Anthropometric measurements were taken with shoes and socks removed. Following the completion of the anthropometric measurements, a 30-min RMR assessment was conducted using a Cosmed K4b^2^ or K5 (used in breath-by-breath mode to align with the K4b^2^) portable indirect calorimeter before participants engaged in activities of their choice for up to 2 hours. In some cases, the RMR assessment was conducted on a separate visit (for a total of 3). Participants were compensated US $25 for the RMR assessment and US $25 for the free-living data collection period.

For the second study visit, an alternative environment was sought for each participant. When this was not possible, participants were asked to engage in different activities than what they chose during their initial visit, which helped to mimic different environments and promote engagement in a wide range of activity behaviors. Similar to the first visit, the participants engaged in behaviors of their choice for up to 2 hours. An additional US $25 of compensation was given for completing the second study visit.

Throughout this study’s visits, participants were given free rein to select activities, so long as they had parental permission, did not put the equipment at risk (eg, from water damage), and did not risk harming themselves or others.

All participants wore the same research-grade devices as described in the equipment portion of part 1 including the primary devices of interest (ActiGraph GT9X on the hip, both wrists, and both ankles; see also Section S1.1 in Multimedia Appendix 1) and the secondary research- and consumer-grade monitors (see Sections S1.2 and S1.3 in Multimedia Appendix 1, respectively). Participants wore a Cosmed K4b^2^ or K5 portable indirect calorimeter, both of which collected breath-by-breath data as a criterion measure of EE (see Section S1.5 in Multimedia Appendix 1 for more detail). During the consent process, participants were able to opt-in to be video recorded throughout each research visit, and only 3 participants did not do so. Live coding was performed for those who did not opt into the video recordings. For the others, the video recordings were later used for direct observation to obtain a record of the behaviors and postures they engaged in, along with relevant contextual factors (eg, indoors versus outdoors and alone versus with others). These video-based observations were performed using the Noldus Observer XT software program for Windows desktop computers.

#### Unstructured Free-Living Study Equipment

Most equipment matched what was used for part 1 of the FLPAY study, with the addition of the Cosmed K5 alongside the K*4b^2^*. The main difference in part 2 was the coding scheme used for direct observation and the reliance on video recordings for direct observation rather than live-coded observations. For participants who did not opt into video recording, real-time observations were performed using similar procedures to those described in part 1 above, except that a new coding scheme was used (Table S2 in Multimedia Appendix 1). Otherwise, the video recordings were coded using the Noldus Observer XT Direct Observation software program (version 12.5) for Windows desktop machines.

The coding scheme for part 2 included a longer list of potential activities and postures, as discussed below. It also included prompts about the activity context (eg, indoor or outdoor and alone or with others). Otherwise, the general structure of the coding scheme matched with part 1, such that every second of the data collection period could be mapped to a single entry in the observation record.

Table S4 in Multimedia Appendix 1 lists the main activities that were included in the coding scheme. As before, extra labels were included for coding the start of the observation period, transition behaviors, and unknown activities (ie, activities not previously defined), and events where the participant was off-camera (ie, in a private space, bathroom, etc). After the primary activity behavior was coded, posture was coded as lying, sitting, standing, standing or stepping, squatting or kneeling, reclining, mixed posture, or unknown (used only when the activity behavior was “off camera”). Section S1.5 in Multimedia Appendix 1 describes the procedures that were used when syncing data from the various sources.

#### Unstructured Free-Living Study Processing of Criterion Data

The data processing procedures for part 2 broadly mirrored what has already been discussed in part 1 resulting in a second-by-second dataset with information about EE, behavior, posture, and context. The direct observation procedures differed from part 1 due to the open-ended nature of this study and the reliance on video recordings. Specifically, at least 2 trained reviewers coded each video, and a third senior reviewer then compared their observations side-by-side. Additional detail is available in S1.4.3 in Multimedia Appendix 1.

## Results

The FLPAY study was funded in June 2016, and recruitment and data collection for part 1 began in January 2017. Funding ended in March 2019, and the project and data collection concluded in March 2020. Some preliminary and auxiliary findings have been reported previously [26,29-31], whereas the protocol described herein will serve as the authoritative guide for ongoing research with the finalized dataset. Part 1 of this study enrolled 100 individuals, and part 2 enrolled 84 individuals. Data loss is described in Section S3 in Multimedia Appendix 1. The overall sample characteristics are shown in Table 1 and Table 2, whereas sample sizes for individual analyses may differ depending on overlap with additional missing data for different activity monitors. Table 3 provides an overview of prior completed analyses and current and ongoing analyses.

**Table 3:** Summary of research activities related to the FLPAY^a^ study.

Component			Notes
Completed analyses			Please see references [26,29-31].
**Ongoing analyses**			
	ActiGraph, GENEActiv, and Axivity	Please see references [36-38]. Ongoing analyses include the development and testing of models to predict energy expenditure [36,37] and classify activity type [38]. Additionally, we are preparing the dataset such that it can be used for out-of-sample validation of models developed by other research groups.	
	activPAL	Please see reference [39].	

^a^FLPAY: Free-Living Physical Activity in Youth.

## Discussion

### Contributions

The design of the 2-part FLPAY study emphasized naturalistic behaviors and periods of transition between activities. This filled an important gap in prior development studies focused on physical behavior characterization. In this paper, we summarized the FLPAY procedures and participants, along with details about criterion datasets that will be useful in future studies that look at various aspects of the data from the activity monitors. Future studies can proceed with novel method development and testing for a variety of research-grade and consumer-grade wearable devices. These data may be especially useful for ongoing work to refine methods involving transition detection for data segmentation [27,30].

It is worth noting that the main device from this study (ActiGraph GT9X) has been replaced by a newer model (ActiGraph LEAP). Although the manufacturer remains committed to backward compatibility, testing will be necessary to compare outputs from the GT9X and LEAP. Such comparisons should include not only direct factors such as agreement of the accelerometer and gyroscope outputs but also indirect factors such as battery life, memory consumption, and participant acceptability. Unpublished data from our laboratory showed that the GT9X battery life was <36 hours when collecting data from both the accelerometer and gyroscope. The LEAP includes a different gyroscope sensor that may be longer-lived, and it also includes additional sensors for richer monitoring (eg, barometer and microphone). These factors may affect usability in lengthy protocols (eg, >1 week) and vary from study to study depending on what sensor configuration is selected. It will also be important to consider the volume of data collected by LEAP, as our unpublished data showed that the GT9X was able to generate >1 GB of data before depleting its battery. As future studies begin to draw on the wearable data from the FLPAY study (especially, but not exclusively, the ActiGraph GT9X data), the implications of these decisions will be important to consider.

### Strengths and Limitations

The FLPAY study had notable strengths, including its collection of data from both laboratory and free-living contexts, with realistic transition periods and behavioral patterns. Another strength was the inclusion of criterion measures for EE, behavior, and posture. These will substantially benefit future work toward the development and validation of novel methodological approaches for the variety of research-grade devices that were used in this study.

Despite its strengths, the FLPAY study also had limitations. A key limitation was the limited diversity of the samples along the lines of racial and ethnic background. While the recruitment strategy was designed to mirror the census makeup in the surrounding area, results may not be fully generalizable for youth who come from underrepresented backgrounds. The open-ended nature of the free-living component could also be viewed as both a strength and a limitation, as the heterogeneity of the data may create a high level of variability that is difficult to address or interpret in future analyses. Lastly, we emphasized detailing the criterion data measurements of RMR, EE, and direct observation in the FLAY study as these will be foundational for a wealth of secondary research that is possible when paired with the monitor data collected in this study.

## Appendix

Multimedia Appendix 1 Additional digital content.

## Abbreviations

EE: energy expenditure
FLPAY: Free-Living Physical Activity in Youth
METy: youth metabolic equivalent
RMR: resting metabolic rate
VO2: oxygen consumption

## Data Availability

Deidentified data from the FLPAY study may be available upon reasonable request from the corresponding author. Video data cannot be deidentified and cannot be shared under any circumstances.
