# Supplementary material for: Calibration and Validation of Machine Learning Models for Physical Behavior Characterization: Protocol and Methods for the Free-Living Physical Activity in Youth (FLPAY) Study
Source: JMIR Res Protoc. 2025 Apr 16;14:e65968. doi: 10.2196/65968 (PMC12044308; doi:10.2196/65968)
Supplement: Multimedia Appendix 1 [file resprot_v14i1e65968_app1.docx]

**Contents**

[S1. Equipment and Measures 6](#_Toc189470972)

[S1.1. Primary Research-Grade Monitor 6](#_Toc189470973)

[S1.1.1 ActiGraph GT9X 6](#_Toc189470974)

[S1.2 Secondary Research-Grade Monitors 6](#_Toc189470975)

[S1.2.1 Axivity AX3 7](#_Toc189470976)

[S1.2.2 GENEActiv 7](#_Toc189470977)

[S1.2.3 ActivPAL^3^ 7](#_Toc189470978)

[S1.3 Secondary Consumer-Grade Monitors 7](#_Toc189470979)

[**Table S1** 9](#_Toc189470980)

[S1.4. Direct Observation Platforms 10](#_Toc189470981)

[S1.4.1. Noldus Observer XT 10](#_Toc189470982)

[**Table S2.** Snapshot of similarities and differences in the direct observation procedures for part 1 versus part 2 of the FLPAY study. 11](#_Toc189470983)

[S1.4.2. Noldus Pocket Observer 12](#_Toc189470984)

[**Table S3** 13](#_Toc189470985)

[S1.4.3. Offline Coding from Video Observations 15](#_Toc189470986)

[**Table S4**. List of Study 2 activities and their operational definitions. 16](#_Toc189470987)

[S1.5. Indirect Calorimetry 19](#_Toc189470988)

[S1.5.1. Cosmed K4b^2^ 19](#_Toc189470989)

[S1.5.2. Cosmed K5 19](#_Toc189470990)

[S1.6. Time Sync Procedures 20](#_Toc189470991)

[S2. Metabolic Calculations and Data Processing 20](#_Toc189470992)

[S2.1. Manufacturer-Specified VO2 Averaging Procedure 20](#_Toc189470993)

[S2.2. Calculating RMR and Cleaning the Data 21](#_Toc189470994)

[S2.3. Calculating Continuous EE 21](#_Toc189470995)

[S3. Data Loss 22](#_Toc189470996)

[**Table S5** 22](#_Toc189470997)

[S3.1. Part 1 (Laboratory Protocol) 22](#_Toc189470998)

[S3.1.1. Direct Observation 23](#_Toc189470999)

[S3.1.2. Indirect Calorimetry 23](#_Toc189471000)

[S3.1.3. Primary Research-Grade Monitor 23](#_Toc189471001)

[**Table S6** 25](#_Toc189471002)

[**Table S7** 26](#_Toc189471003)

[S3.1.4. Secondary Research- and Consumer-Grade Monitors 27](#_Toc189471004)

[S3.2. Part 2 (Free-Living Protocol) 27](#_Toc189471005)

[S3.2.1. Direct Observation 27](#_Toc189471006)

[S3.2.2. Indirect Calorimetry 27](#_Toc189471007)

[S3.2.3. Primary Research-Grade Monitors 27](#_Toc189471008)

[S3.2.4. Secondary Research- and Consumer-Grade Monitors 27](#_Toc189471009)

[**Table S8** 29](#_Toc189471010)

[References 30](#_Toc189471011)

# S1. Equipment and Measures

## S1.1. Primary Research-Grade Monitor

### S1.1.1 ActiGraph GT9X

*.* The GT9X is a small (3.5 x 3.5 x 1 cm), lightweight (14 g), water resistant (1 meter, 30 minutes) research-grade activity monitor that can be worn on the hip, wrist, or ankle. The GT9X contains a programmable display window and a variety of sensors. The first sensor is the primary triaxial accelerometer, which has a dynamic range of ± 8 gravitational units (*g*) and can be initialized to collect data at sampling frequencies of 30-100 Hz in increments of 10 Hz. Additional sensors are housed in the inertial measurement unit (IMU), all of which sample at a non-modifiable rate of 100 Hz. The IMU houses a secondary triaxial accelerometer with a dynamic range of ± 16 *g*’s, a triaxial gyroscope with a dynamic range of ± 2000 degrees per second, a magnetometer with a dynamic range of ± 4800 micro-Tesla, and a thermometer that measures the temperature inside the device housing. For the present study, GT9X devices were initialized for the primary accelerometer to collect data at 90 Hz, with the full IMU enabled. The 90-Hz setting was chosen based on prior recommendations to select a multiple of 30 Hz when using ActiGraph devices, although this seems to primarily matter for conversion to counts and does not affect the raw acceleration data [1].

## S1.2 Secondary Research-Grade Monitors

Axivity AX3 (Axivity Ltd., Newcastle, UK) and GENEActiv (ActivInsights Ltd. Kimbolton, Cambridgeshire, UK) were worn on opposite wrists, with half of participants wearing the GENEActiv on the left wrist and AX3 on the right wrist, while the other half did the opposite. The AX3 and GENEActiv were positioned proximal to the wrist worn ActiGraph GT9X devices and affixed using the respective watch bands supplied by the manufacturers.

Participants also wore two activPAL^3^ devices (PAL Technologies Ltd., Glasgow, Scotland), with one on the anterior aspect of each thigh at the midpoint between the iliac crest and the top of the patella. The activPAL^3^ devices were wrapped in parafilm to cover the micro-USB port and then attached to the thigh using Tegaderm transparent adhesive film dressing (3M, Saint Paul, MN, USA).

### S1.2.1 Axivity AX3

*.* The AX3 is a small (23.0 x 32.5 x 7.6 mm) and lightweight (11 g) research-grade activity monitor that can be worn at multiple attachment sites, of which the wrist is the most commonly used. The AX3 contains a triaxial accelerometer that is user-configurable, with available sampling frequencies between 12.5-3200 Hz and user-selectable dynamic ranges of ± 2, 4, 8, or 16 *g’s*. For the present study, AX3 devices used were configured to collect raw acceleration data at 100 Hz with a dynamic range of ± 8 *g*’s.

### S1.2.2 GENEActiv

*.* The GENEActiv is a small (43 x 40 x 13 mm), lightweight (16 g), and waterproof research-grade activity monitor that is typically worn on the wrist or hip, although it is possible to use other attachment sites. The GENEActiv contains a triaxial accelerometer with a dynamic range of ±8 *g* and a user-configurable sampling frequency (10-100 Hz, in increments of 10) as well as ambient light and temperature sensors. For the present study, GENEActiv devices used were initialized to collect at 100 Hz.

### S1.2.3 ActivPAL^3^

*.* activPAL^3^ devices (PAL Technologies. Glasgow, Scotland, UK) are small (23.5mm x 43mm x 5mm), lightweight (9.5g) devices designed to be worn on the thigh. The activPAL^3^ devices contain a triaxial accelerometer that collects at a sampling frequency of 20 Hz and has a dynamic range of ± 2 *g*’s.

## S1.3 Secondary Consumer-Grade Monitors

The secondary monitors included consumer-grade options as an exploratory outcome, as previously presented [2]. These included the following: Apple Watch 2 (Apple Inc., Cupertino, CA, USA – worn on the left wrist, next to the assigned GENEActiv or AX3 device and approximately 4” proximal to the styloid process); Mymo activity tracker (formerly available from TupeloLife Services LLC, Dallas, TX, USA – attached next to the hip-worn GT9X device on the same elastic belt, approximately 1” lateral of the iliac crest on the right hip); Misfit Shine 2 device (formerly available from Misfit Wearables Inc., Burlingame, CA, USA – attached next to the Mymo on the elastic belt, approximately 2” lateral of the iliac crest of the right hip); and a second Misfit Shine 2 (attached to the right shoelace using the manufacturer supplied clip). Subsets of participants were also assigned to wear either a Fitbit Charge 2 (manufactured by Fitbit LLC, San Francisco, CA, USA) or Samsung Gear Fit2 (Samsung Group, Seoul, South Korea), with the assigned device being worn on the right wrist, adjacent to the GENEActiv or AX3 monitor and roughly 4” proximal to the styloid process).

Table S1 summarizes the secondary consumer-grade devices that were used in this study. Results for energy expenditure have been reported previously [2]. All five consumer-grade devices used in the FLPAY study had an accompanying smartphone application or website for device setup and data syncing. These interfaces generally required inputs such as the participant’s age, biological sex, height, and weight. After entering the required information, the devices were individually synchronized to mobile devices (Apple iPhone 5S or Samsung Galaxy S6), marking the start of data collection.

### **Table S1.** Consumer-grade monitors used in part 1 of the study.

| **Device** | **Website** | **Outcomes Available** | **Current Model** |
| --- | --- | --- | --- |
| **Apple Watch 2** | https://www.apple.com/newsroom/2016/09/apple-introduces-apple-watch-series-2/ | Steps, kcals (gross and net active), active minutes, distance | Apple Watch Series 9 |
| **Fitbit Charge 2** | https://www.fitbit.com/charge2/charge2-101 | Steps, kcals (gross), distance, flights of stairs, active minutes | Fitbit Charge 6 |
| **Misfit Shine 2** | [Device Discontinued] | Steps, kcals (gross), distance, proprietary “points” | N/A |
| **Mymo Activity Tracker** | [Device Discontinued] | Steps, kcals (gross), distance, active minutes | N/A |
| **Samsung Gear Fit2** | https://www.samsung.com/us/mobile/wearables/smart-fitness-bands/samsung-gear-fit2-large-black-sm-r3600daaxar/ | Steps, kcals (gross), flights of stairs | Samsung Galaxy Fit 3 |

## S1.4. Direct Observation Platforms

All video recordings were made using a Canon Vixia HFR700 camcorder.

### S1.4.1. Noldus Observer XT

*.* The Noldus Observer XT (version 12.5) is a computer software program designed for direct observation on desktop machines. Users create a “coding scheme” that is specific to the study and dictates the format of data output. The coding scheme can be exported to the Pocket Observer application (discussed below) for real-time coding via a mobile device. For the FLPAY study, coding schemes for part 1 and part 2 were developed such that only one behavior and posture could be selected at a time. (The same applied for the additional modifiers in part 2, such as context and company.) Coding was done continuously via focal sampling, resulting in a continuous record of behavior and posture. For part 1 of the study (laboratory setting), the Pocket Observer application was used, while for part 2 (free-living settings) the desktop application was used. A snapshot of the similarities and differences in the direct observation procedures for the two study components is detailed in Table S2.

**Table S2.** Snapshot of similarities and differences in the direct observation procedures for part 1 versus part 2 of the FLPAY study.

|  | **Part 1** | **Part 2** | **Operational Definition of Posture** |
| --- | --- | --- | --- |
| **Direct Observation Collection Methods** | | | |
| Primary collection method | Live coding | Video recording |  |
| Secondary collection method^1^ | Video recording | Live coding^2^ |  |
| **Coding Scheme Characteristics** | | | |
| Activities coded | See Table S3 | See Table S4 |  |
| Postures coded | Lying | Lying | lying on their back in a supine position |
|  | Sitting | Sitting | sitting upright |
|  | Standing | Standing | standing upright |
|  | Reclining | Reclining | sitting in a reclined position, but not supine |
|  | Stepping | Stepping | standing upright and taking steps |
|  | - | Squatting/Kneeling | Used when participant is not seated or standing upright but in a squat or kneel or combination of both |
|  | - | Mixed Posture | Used when the behavior is continuous, but the posture is changing, and no definable posture can be assigned. |
|  | Unknown | Unknown | Used when Off Camera |
| Environments coded | N/A | Indoor, outdoor |  |
| Company coded | N/A | Alone, with peers, with adults, etc. |  |
| ^1^Included as a backup and failsafe if anything failed with the primary collection method ^2^Used for participants who did not opt in to video recording | | | |

### S1.4.2. Noldus Pocket Observer

**.** This mobile application was used for all laboratory visits and any free-living visits for which participants did not opt into video recording. For part 1 of the study, the Pocket Observer interface allowed users to indicate a new behavior by tapping the corresponding box on the screen (one box for each of the 16 prescribed behaviors, plus two more for ‘Transition’ and ‘Unknown’) then tapping a subsequent pop-up to indicate the posture (lying, sitting, reclining, standing, stepping, or unknown) once the primary activity behavior label had been coded (Table S3). The default behavior at the start of each recording was labeled ‘CommentStartTime’, prompting users to enter the Windows system clock start time as a comment that allowed subsequent syncing with other devices (see section 1.5). The Android system tracked the timing of each button press, and the coding scheme was set up so that behaviors were continuous (i.e., no gaps allowed) and mutually exclusive (i.e., no multi-tasking).

### **Table S3**

. Operational definitions and activity labels for activities performed during the laboratory study protocol. The sixteen activity labels were the focus of direct observation during data collection, whereas the 6- and 14-activity reclassifications were applied post hoc, as needed for specific analyses.

| **Activity Group Labels (n = 6)** | **Individual Activity Class Labels (n = 14)** | **Activity Labels (n = 16)** | **Operational Definition of Activity** |
| --- | --- | --- | --- |
| **Seated/Lying Activity** | Lying | Lying | Lying supine with arms by the side in a quiet room with no stimuli |
|  | Computer | Computer Gaming | Sitting at a tabletop or desk playing a self-selected computer game (e.g., Tetris, slither.io, etc.) |
|  |  | Internet Browsing | Sitting at a tabletop or desk using the internet to browse websites and perform computer-based tasks (e.g., e-mail, reading, typing, etc.) |
|  | Reclining | Reclining | Sitting in an office chair in a reclined posture |
|  | Reading | Reading | Reading a physical book |
| **Chores** | Dusting | Dusting | Wiping down a tabletop or desk surface with a paper towel and spray bottle |
|  | Sweeping | Sweeping | Sweeping paper shreds with a broom and dustpan from a hallway floor |
| **Continuous Walking and Running** | Overground Walking | Slow Overground Walking | Walking at a self-selected slow and leisurely pace around the perimeter of a basketball or tennis court |
|  |  | Brisk Overground Walking | Walking at a self-selected brisk pace around the perimeter of a basketball or tennis court as if they were in a hurry or running late for something |
|  | Overground Running | Overground Running | Running at a self-selected pace that could be maintained for approximately 5 minutes around the perimeter of a basketball or tennis court |
| **Sports** | Catch | Catch | Passing a football back and forth with a partner at a self-selected comfortable distance |
|  | Soccer | Soccer | Simulated soccer drills (e.g., one-vs-one gameplay, dribbling, passing, shooting, defending, etc.) |
|  | Basketball | Basketball | Simulated basketball drills (e.g., one-vs-one gameplay, dribbling, passing, shooting, defending, etc.) |
| **Wheels** | Stationary Cycling | Stationary Cycling | Riding a stationary cycle ergometer at a self-selected resistance and cadence (RPM) |
| **Recreation and Leisure Time Activity** | Stair Walking | Stair Walking | Ascending and descending a staircase at a self-selected pace |
|  | Jumping Jacks | Jumping Jacks | Continuous self-paced jumping jacks |

### S1.4.3. Offline Coding from Video Observations

**.** Due to the increased complexity of free-living behavior compared to the laboratory protocol, a more general coding scheme was designed for part 2 (Table S4). Emphasis was placed on coding video recordings, rather than the real-time live-coded approach that was taken for the simpler protocol in part 1. All videos for part 2 were coded by two independent trained reviewers who completed training by reaching agreement ≥ 80% with criterion training videos. Inter-observer agreement was calculated using the Noldus Observer XT software. Observations were time aligned and then compared using a ‘frequency/sequence’ approach (i.e., considering the timing, number of occurrences, and order of events) with a 1.0 second tolerance window. Following initial review with these settings, mean (± SD) agreement was 47.9% ± 24.9% between coders. These coded observations were compared side-by-side by a senior reviewer and a harmonized version was created to resolve discrepancies between reviewers with preference given to the coded observation with more detail.

**Table S4**. List of study 2 activities and their operational definitions.

| **Activity** | **Operational Definition** |
| --- | --- |
| Arts/Crafts | behavior may include drawing, painting, coloring, sketching, Playdough, clay, etc. |
| Baseball/Softball | playing an organized baseball/softball game |
| Baseball - Drills | simulated baseball/softball tasks such as swinging a bat, hitting, catching, pitching, fielding, base running, etc. |
| Basketball | playing basketball or simulated basketball game scenario against an opponent |
| Basketball - Drills | playing a game with a basketball and hoop (e.g., Horse), general shooting around, dribbling, etc. |
| Reading | reading or interacting with a physical book |
| Blocks | includes Legos, building block, K'nex, Lincoln Logs, Magnetic building sets, etc. |
| Card/Board Games | includes playing card games and board games such as Uno, Monopoly, Life, Sorry, etc. |
| Catch | playing catch with a ball, not associated with playing the sport the ball is intended for, just throwing the ball back and forth in a pair or group |
| Cell Phone | general use of a cell phone includes social media, games, reading, texting, e-mailing, calling, etc. |
| Cleaning/Wiping | involves using a cloth or rag to dust or wipe down windows, tabletops, etc. |
| Computer | general use of a desktop computer with a mouse. Includes social media, viewing videos, doing homework, e-mailing, reading, etc. |
| Cooking | includes cooking, baking, food prep tasks in the kitchen |
| Dishes | includes loading and unloading he dishwasher as well as washing and drying dishes by hand |
| Dog Walking (on a leash) | continuous overground walking with a dog on a leash |
| Football | playing a game of touch football |
| Football - Drills | simulated football includes running, passing, catching, kicking, etc. Primarily in an individual or pair |
| Gardening | general gardening tasks including weeding, planting, picking fruit/vegetables, etc. |
| Golf | playing a round of golf |
| Golf - Drills | simulated golf tasks including swinging a golf club and hitting a golf ball, putting, driving range, etc. |
| Homework - Screen | general button for tasks involving mixed homework activities using a combination of a screen and pencil/pen and paper and handwritten activities or book reading and digital response/interaction with a screen |
| Homework - Writing | general button for tasks involving writing on paper by hand and reading a book without a screen |
| Laptop | general use of a laptop computer. Includes social media, viewing videos, doing homework, e-mailing, reading, etc. |
| Laundry | includes folding and putting away laundry |
| Lying Only | to be used when lying is the primary activity behavior. Not to be used when the participant is sitting and engaging with something else (e.g., watching TV, using cellphone, viewing laptop, etc.). |
| Mowing - Push | cutting the grass, pushing a lawnmower |
| Mowing - Riding | cutting the grass, riding a lawnmower |
| Overground Cycling | riding a bicycle outdoors, over ground |
| Playground - Climbing | climbing on gym or playground equipment |
| Playground - Other | general button for playground activities involving a jungle gym or similar type equipment. This is a general, non-specific button to be used when the participant is on the playground but not engaging in a discernable activity |
| Playground - Swing | includes swinging on a standard playground swing or tree swing |
| Playground - Slide | involves the participant using the slide, as it is intended, for sliding down from the top to the bottom |
| Raking | raking leaves or sand/dirt |
| Running - Treadmill | continuous running on a treadmill |
| Running (Overground) | continuous running over ground |
| Scooter | riding a self-propelled scooter over ground |
| Sitting Only | to be used when sitting is the primary activity behavior. Not to be used when the participant is sitting and engaging with something else (e.g., watching TV, using cellphone, viewing laptop, etc). |
| Roller Skating | includes in-line skating, rollerblading, etc. |
| Skateboarding | riding a skateboard |
| Soccer | playing a game of soccer or simulated soccer game activities against an opponent. Includes running, kicking, defending, shooting, passing, etc. |
| Soccer Drills | passing, shooting, dribbling, juggling tasks, primarily as an individual or in a pair |
| Stair Climbing | ascending and descending stairs. To be used when stair climbing is a continuous activity and not when occurring as a transition between activities |
| Sweeping/Mopping | sweeping or mopping the floor with a broom or mop or another floor cleaning device (e.g., Swiffer) |
| Tablet | general use of a table - includes social media, viewing videos, doing homework, e-mailing, reading, etc. |
| Tag/Hide & Seek | playing a game of tag or hide & seek |
| Tennis | playing tennis with an opponent |
| Tennis - Drills | simulated tennis drills include serving, returning, and retrieving balls on a tennis court. Can be done individually |
| Transition | intended to be used when there is a clear break from one activity before beginning the next activity. Note: exercise good judgement and caution when using this label. |
| Unknown Activity | intended to be used when an activity/behavior occurs that is not included or defined in this list. Note: be sure to carefully review this list before using this button to make sure what you are coding is not already defined in another behavior description |
| Video Gaming | includes using a controller to play a game on a screen (TV, computer, etc. |
| Volleyball | playing volleyball or simulated volleyball with teammates and an opponent |
| Volleyball - Drills | simulated volleyball drills include serving, returning, and retrieving balls on a volleyball court. Can be done individually |
| Walking (Treadmill) | continuous walking on a treadmill |
| Walking (Overground) | continuous walking over ground |
| Watching TV | viewing a TV on a TV not watching video on another screen type |

## S1.5. Indirect Calorimetry

### S1.5.1. Cosmed K4b^2^

*.* The Cosmed K4b^2^ (Cosmed S.r.l., Rome, Italy) served as a criterion measure of energy expenditure (EE) for all FLPAY participants in part 1 and a subset of participants in part 2. It is a portable indirect calorimeter (170 x 55 x 100mm; 800 g) worn on the torso in a harness that holds the analyzer unit and battery pack. Participants wear a silicon facemask that is held in place using snap-on adjustable nylon fabric headgear, creating a seal around the mouth and nose. The facemask is fitted with both a turbine flowmeter and a sampling line, which together allow breath-by-breath measurement of ventilation and respiratory exchange of oxygen (O_2_) and carbon dioxide (CO_2_). Prior to each test, a four-stage calibration was performed, first to calibrate the gas analyzers to room air (20.93% O_2_; 0.03% CO_2_), then to calibrate them to a known gas mixture (15.98% O_2_; 4.008% CO_2_; balance nitrogen), then to calibrate the flowmeter to a known volume (drawn from a 3-L Hans Rudolf syringe), and finally to calibrate the system for synchronizing data from the flowmeter and gas analyzers (i.e., to account for the delay incurred by pumping breath samples to the analyzers). The K4b^2^ has been previously validated against the gold-standard Douglas Bag method for measuring O_2_ consumption and CO_2_ production [3].

### S1.5.2. Cosmed K5

*.* The Cosmed K5 (Cosmed S.r.l., Rome, Italy) is a newer portable indirect calorimeter which functions similarly to the K4b^2^, measuring O_2_ consumption and CO_2_ production. It served as a criterion measure of EE for the majority of participants in part 2 of the FLPAY study. It is small and lightweight (174 x 111 x 64mm; 900 g) and designed to be worn in a backpack-style harness that holds the analyzer unit. The K5 has an interactive touchscreen LCD display (3.5in) and is capable of transmitting data via USB and Bluetooth and has a listed on-board memory capacity of 2,048,000 breaths [4]. Participants wear the same silicon facemask contraption described previously, and the K5 device can be configured to collect data using either breath-by-breath or mixing chamber (1 cm^3^) sampling methods. The former was used for the present study, to ensure comparable data collection from the K5 and K4b^2^. Prior to each use, the K5 was calibrated using the same steps described for the K4b^2^, plus an additional scrubber calibration (for indoor tests) which zeroed the CO_2_ analyzer by passing sampled room air through a column of soda lime to remove the CO_2_. Reference gas for the K5 was nitrogen-balanced with 16.00% O_2_ and 5.00% CO_2_. The K5 has been previously validated against the gold-standard Douglas Bag method for measuring O_2_ consumption and CO_2_ production [4].

## S1.6. Time Sync Procedures

For both part 1 and part 2 of the study, all data were synced to a Windows system clock. The ActiGraph devices were initialized on a Windows machine and automatically synced to its clock via the Actilife software. For the Cosmed and Noldus devices, the research team started each recording while viewing a Windows clock to obtain a known start time. This served as a reference point for converting relative timestamps in the data files (e.g., 30 seconds since the start of the recording) into Windows-equivalent timestamps. Similarly, for the video recordings, when the system clock was filmed, timestamps could be calculated relative to that point in the video recording.

# S2. Metabolic Calculations and Data Processing

## S2.1. Manufacturer-Specified VO2 Averaging Procedure

For assessments of resting metabolic rate (RMR) and epoch-level oxygen consumption (VO_2_) in both part 1 and part 2, it was necessary to calculate average VO_2_ from breath-by-breath readings. A specialized process was used, which involved first calculating arithmetic means for certain core variables (e.g., gas fractions), then using those values to calculate a single VO_2_ value for the whole averaging period. This approach was suggested by the manufacturer (personal communication) rather than directly averaging the VO_2_ values that the system gave for each breath.

## S2.2. Calculating RMR and Cleaning the Data

Identical procedures were used for determining RMR in both the laboratory and free-living protocols, regardless of whether data came from a Cosmed K4b^2^ (laboratory participants and subsample of free-living participants) or Cosmed K5 (free-living participants only). Breath-by-breath data were extracted from the RMR periods (30 min of supine rest). The first 10 min and final min of data were discarded. For the remaining data, a 5-min rolling average was calculated, using the manufacturer’s averaging method to calculate each value. RMR was then defined as the lowest observed VO_2_ value from the rolling window. For each age group (i.e., ≤ 12 years versus > 12 years), values were removed if they were more than ± 2 SD from the mean. Data were also manually inspected to check for anomalies that were undetected by the prior processes. Any such cases were also removed from the analysis.

## S2.3. Calculating Continuous EE

For both part 1 and part 2 of the study, the VO_2_ data were aggregated into a second-by-second readout of EE. To do this, breath-by-breath data were first averaged every 15 seconds using the procedure described above in section 2.1. The resulting VO_2_ values were then divided by RMR to obtain EE (MET_y_). Lastly, each value was copied 15 times (once for each second in the epoch), resulting in the second-by-second output.

# S3. Data Loss

Table S5 shows participant- and visit-level data loss for both parts of the FLPAY study. Overall, 99 out of 100 participants provided at least some data in part 1, while 83 out of 84 did so in part 2. Most participants in either part completed both days of data collection. The main cause of incompletion in part 1 was withdrawal, while in part 2 there was a mix of withdrawal and premature study termination due to the COVID-19 pandemic.

### **Table S5.** Sample sizes for part 1 and part 2 of the FLPAY study, accounting for participant- and visit-level data loss.

|  | **Days of Data** | | |
| --- | --- | --- | --- |
|  | **Zero^a^** | **One^b^** | **Two^c^** |
| **Part 1 (n)** | 1 | 6 | 93 |
| **Part 2 (n)** | 1 | 5 | 78 |
| ^a^Represents total incompletion of the data collection protocol (e.g., exclusion or withdrawal before collection)  ^b^Represents partial completion of the data collection protocol (e.g., withdrawal after the first visit)  ^c^Represents full completion of the data collection protocol | | | |

Apart from participant- and visit-level data loss, miscellaneous additional data loss occurred for a variety of reasons, some affecting a specific device (e.g., if the participant refused to wear the Cosmed), others affecting a specific part of the protocol (e.g., if a laboratory activity had to be skipped due to space unavailability), and still others affecting both (e.g., if a certain monitor became dislodged during an activity, and could not be reattached until completion of the activity). Accordingly, future analyses using FLPAY data may have differing sample sizes, depending on which devices and protocol segments are being analyzed. This will require study-specific data accounting. Nevertheless, we provide a general description of additional data loss below.

## S3.1. Part 1 (Laboratory Protocol)

The expected sample size from each source was 99, consistent with Table S5.

### S3.1.1. Direct Observation

*.* Complete or partial data were available for all 99 participants, totaling 228 hours. The main cause of data loss was withdrawal after the first day of data collection, while other causes occurred more rarely (e.g., refusal to complete a specific activity, or unavailability of the required space). Of the 99 participants with data, 58% completed two bouts of all 16 activities, while 83% completed at least one bout of all 16 activities. Further loss was minimal, with 91% of participants completing two bouts for ≥14 activities.

### S3.1.2. Indirect Calorimetry

*.* There were 85 participants with continuous VO_2_ measurement for all or part of the activity protocol, totaling 190 hours. The remaining 14 participants were missing or removed for the following reasons: did not perform the RMR assessment (n = 3); refused to wear the Cosmed after completing the RMR assessment (n = 3, one of whose RMR data were not saved for outlier analysis); RMR outliers, i.e., values >2 SD above or below the age-group mean (n = 6); and implausible RMR data after manual inspection, e.g., excessive breath-by-breath variability potentially indicating device malfunction (n = 2). The reason that RMR-related issues caused loss of all indirect calorimetry data was that MET_y_ could not be calculated without a valid RMR measurement, as described in the main document.

Apart from continuous VO_2_ measurement, a separate dataset was assembled for steady state periods, as described in the main document. The latter dataset was subjected to further cleaning, which is summarized in Table .

### S3.1.3. Primary Research-Grade Monitor

*.* Table S7 summarizes sample size for the primary research-grade activity monitor. ActiGraph GT9X data were generally collected from both the primary accelerometer and the inertial measurement unit. No participants were missing primary accelerometer data. The inertial measurement unit was not properly activated in five cases, all of which were separate participants (one day apiece).

### **Table S6.** Summary of missing Cosmed data from steady state periods.

| **Description** | **Bouts lost (n)** | **Remaining Steady State Bouts (n)** |
| --- | --- | --- |
| Expectation (16 steady state bouts * 99 participants) | -- | 1584 |
| **Participant-level Data Loss (n = 15)** |  |  |
| Did not perform RMR assessment (n = 3) | 48 | 1536 |
| RMR outliers (n = 6) | 96 | 1440 |
| RMR data removed after manual inspection (n = 2) | 32 | 1408 |
| Refused K*4b^2^* after completing RMR (n = 3)^*^ | 48 | 1360 |
| **Day-level Data Loss** |  |  |
| Withdrew after first day (n = 6) | 48 | 1312 |
| **Activity-level Data Loss: ^K^*^4b2^* Data Cleaning** |  |  |
| Activity duration < 3 minutes, 40 seconds | 68 | 1244 |
| MET_y_ outliers | 53 | 1191 |
| Missing or incomplete data (e.g., battery died during activity) | 20 | 1171 |
| **Activity-level Data Loss: Other** |  |  |
| Failure/refusal to perform/complete an activity | 3 | 1168 |
| Basketball court unavailable | 8 | 1160 |

RMR- resting metabolic rate.

^*^ For one RMR-only participant, the data were not saved. For the other two, the data were saved and therefore still included in the RMR analysis (i.e., to calculate outliers) despite having no additional data available for the 16 activity bouts.

### **Table S7.** Available data for the primary research-grade wearable activity monitors (ActiGraph) in part 1 of the FLPAY study (expected n = 99, per Table S2). Notably, data loss sometimes affected all monitors equally (e.g., due to participant withdrawal), and other times affected only certain ones (e.g., due to download error).

|  | **Days of Data** | | |
| --- | --- | --- | --- |
|  | **Zero** | **One** | **Two** |
| **ActiGraph (n)^a^** |  |  |  |
| Hip | 0 | 8 | 91 |
| Left Wrist | 0 | 10 | 89 |
| Right Wrist | 0 | 8 | 91 |
| Left Ankle | 0 | 8 | 91 |
| Right Ankle | 0 | 8 | 91 |
| ^a^All participants wore several of these monitors (i.e., one at each of the listed attachment sites) | | | |

### S3.1.4. Secondary Research- and Consumer-Grade Monitors

*.* Data were mostly complete for the secondary research-grade devices; however, data loss was common for consumer-grade activity monitors as they were an exploratory outcome. Sample sizes for consumer-grade devices in part 1 have been reported previously [2].

## S3.2. Part 2 (Free-Living Protocol)

The expected sample size from each source was 83, consistent with Table S5.

### S3.2.1. Direct Observation

*.* Complete or partial data were available for all 83 participants, totaling 303 hours. Three participants did not opt into the video recordings, and thus they were observed by live coding.

### S3.2.2. Indirect Calorimetry

*.* There were 72 participants with continuous VO_2_ measurement for all or part of the protocol, totaling 238 hours. In some cases, the dataset included all or part of the RMR assessment, if it was conducted immediately before the main activity protocol. One participant provided only RMR data, choosing not to continue wearing the Cosmed thereafter. The 11 participants with no continuous VO_2_ data were missing or removed for the following reasons: Refused to wear the Cosmed altogether (n = 2); RMR outliers (n = 3); and errors of data collection or storage, e.g., due to device malfunction or user mistakes (n = 6).

### S3.2.3. Primary Research-Grade Monitors

*.* Table S8 summarizes sample size for the primary research-grade activity monitors (ActiGraph).

### S3.2.4. Secondary Research- and Consumer-Grade Monitors

*.* Data were mostly complete for the secondary research-grade monitors; however, data loss was common for consumer-grade activity monitors, with more than 50 of the 83 participants experiencing an issue for at least one monitor on at least one day. Reasons for data loss ranged from connectivity issues (e.g., problems with mobile hotspot or Bluetooth for syncing estimates between the watch, phone, and mobile application platform) to physical issues (e.g., devices did not fit the participant) and protocol-related challenges (e.g., insufficient time for monitor attachment and syncing, or participant removal of the Cosmed which broke the synchrony between devices). The extensive difficulties with the consumer-grade devices, in conjunction with their exploratory role in the study, thus rendered the data unusable for most purposes.

### **Table S8.** Available data for the primary research-grade wearable activity monitors (ActiGraph) in part 2 of the FLPAY study. In cases where the sample size differs from 5, the participant may have completed the data collection protocol despite losing data from a particular monitor (e.g., due to download error).

|  | **Days of Data** | | |
| --- | --- | --- | --- |
|  | **Zero** | **One** | **Two** |
| **ActiGraph (n)^a^** |  |  |  |
| Hip | 0 | 6 | 77 |
| Left Wrist | 0 | 5 | 78 |
| Right Wrist | 0 | 6 | 77 |
| Left Ankle | 0 | 6 | 77 |
| Right Ankle | 0 | 7 | 76 |
| ^a^All participants wore several of these monitors (i.e., one at each of the listed attachment sites) | | | |

# References

1. Brønd JC, Arvidsson D. Sampling frequency affects the processing of Actigraph raw acceleration data to activity counts. J Appl Physiol (1985). 2016 Feb 1;120(3):362-9. PMID: 26635347. doi: 10.1152/japplphysiol.00628.2015.

2. LaMunion SR, Blythe AL, Hibbing PR, Kaplan AS, Clendenin BJ, Crouter SE. Use of consumer monitors for estimating energy expenditure in youth. Appl Physiol Nutr Metab. 2020 Feb;45(2):161-8. PMID: 31269409. doi: 10.1139/apnm-2019-0129.

3. McLaughlin JE, King GA, Howley ET, Bassett DR, Jr., Ainsworth BE. Validation of the COSMED K4 b2 portable metabolic system. Int J Sports Med. 2001 May;22(4):280-4. PMID: 11414671. doi: 10.1055/s-2001-13816.

4. Crouter SE, LaMunion SR, Hibbing PR, Kaplan AS, Bassett DR, Jr. Accuracy of the Cosmed K5 portable calorimeter. PLoS ONE. 2019;14(12):e0226290. doi: 10.1371/journal.pone.0226290.
